# Unexpected cancer-predisposition gene variants in Cowden syndrome and Bannayan-Riley-Ruvalcaba syndrome patients without underlying germline *PTEN* mutations

**DOI:** 10.1371/journal.pgen.1007352

**Published:** 2018-04-23

**Authors:** Lamis Yehia, Ying Ni, Kaitlin Sesock, Farshad Niazi, Benjamin Fletcher, Hannah Jin Lian Chen, Thomas LaFramboise, Charis Eng

**Affiliations:** 1 Genomic Medicine Institute, Lerner Research Institute, Cleveland Clinic, Cleveland, Ohio, United States of America; 2 Department of Pathology, Case Western Reserve University, Cleveland, Ohio, United States of America; 3 Center for Clinical Genomics, Cleveland Clinic, Cleveland, Ohio, United States of America; 4 Department of Genetics and Genome Sciences, Case Western Reserve University School of Medicine, Cleveland, Ohio, United States of America; 5 Germline High Risk Focus Group, CASE Comprehensive Cancer Center, Case Western Reserve University, Cleveland, Ohio, United States of America; 6 Taussig Cancer Institute, Cleveland Clinic, Cleveland, Ohio, United States of America; St. Jude Children's Research Hospital, UNITED STATES

## Abstract

Patients with heritable cancer syndromes characterized by germline *PTEN* mutations (termed *PTEN* hamartoma tumor syndrome, PHTS) benefit from *PTEN*-enabled cancer risk assessment and clinical management. *PTEN-*wildtype patients (~50%) remain at increased risk of developing certain cancers. Existence of germline mutations in other known cancer susceptibility genes has not been explored in these patients, with implications for different medical management. We conducted a 4-year multicenter prospective study of incident patients with features of Cowden/Cowden-like (CS/CS-like) and Bannayan-Riley-Ruvalcaba syndromes (BRRS) without *PTEN* mutations. Exome sequencing and targeted analysis were performed including 59 clinically actionable genes from the American College of Medical Genetics and Genomics (ACMG) and 24 additional genes associated with inherited cancer syndromes. Pathogenic or likely pathogenic cancer susceptibility gene alterations were found in 7 of the 87 (8%) CS/CS-like and BRRS patients and included *MUTYH*, *RET*, *TSC2*, *BRCA1*, *BRCA2*, *ERCC2* and *HRAS*. We found classic phenotypes associated with the identified genes in 5 of the 7 (71.4%) patients. Variant positive patients were enriched for the presence of second malignant neoplasms compared to patients without identified variants (OR = 6.101, 95% CI 1.143–35.98, p = 0.035). Germline variant spectrum and frequencies were compared to The Cancer Genome Atlas (TCGA), including 6 apparently sporadic cancers associated with PHTS. With comparable overall prevalence of germline variants, the spectrum of mutated genes was different in our patients compared to TCGA. Intriguingly, we also found notable enrichment of variants of uncertain significance (VUS) in our patients (OR = 2.3, 95% CI 1.5–3.5, p = 0.0002). Our data suggest that only a small subset of *PTEN-*wildtype CS/CS-like and BRRS patients could be accounted for by germline variants in some of the known cancer-related genes. Thus, the existence of alterations in other and more likely non-classic cancer-associated genes is plausible, reflecting the complexity of these heterogeneous hereditary cancer syndromes.

## Author summary

In the US, there are 12 million individuals living with heritable cancer, with its burden of morbidity and mortality. Heritable cancers occur when an individual is born with particular genetic errors (germline mutations). Because many germline mutations are known to cause specific cancers, knowing which patient has mutations in what gene allows for the practice of preventative, proactive, rather than reactive, medicine. However, the challenge is that many patients with classic symptoms test negative for mutations in genes known to be associated with the diagnosed clinical syndrome. This is true for Cowden (CS) and Bannayan-Riley-Ruvalcaba (BRRS) syndromes, whereby only a subset of patients have germline mutations in *PTEN*. Due to advances in biomedical research, we now know that there are multiple cancer-related genes besides *PTEN*. Importantly, these genes had not been examined in CS and BRRS patients. Here, we identify such variants in 8% of these patients, with the majority showing clinical features known to be associated with the mutated genes. These findings suggest potentially different clinical management, guided by the specific genes identified. More importantly, the remaining patients who tested negative for the known studied cancer-associated genes suggest yet-to-be discovered genes responsible for CS/BRRS.

## Introduction

Hereditary cancer syndromes account for approximately 5–10% of all cancers, representing the subset for high risk management [1, 2]. Cowden syndrome (CS) and Bannayan-Riley-Ruvalcaba syndrome (BRRS) are good models for heritable cancer. CS is an underdiagnosed difficult-to-recognize autosomal dominant disorder characterized by multiple hamartomas and increased lifetime risks of breast, thyroid and other carcinomas [3, 4]. Furthermore, individual features mimic sporadic disease as well as other syndromes. BRRS is classically characterized by macrocephaly in combination with intestinal hamartomatous polyposis, vascular malformations, lipomas, and genital freckling [5]. Subsets of CS, BRRS, and related disorders characterized by germline *PTEN* mutations are collectively known as *PTEN* hamartoma tumor syndrome (PHTS) [6, 7]. We and others have independently shown that CS-PHTS have elevated lifetime risks of breast (85%), thyroid (35%), renal (33%), endometrial (28%), and colorectal (9%) cancers, and melanoma (6%) [6, 8, 9]. Medical management is therefore based on these *PTEN*-associated cancer risks [10–12].

Clinical diagnostic criteria for CS were developed by the International Cowden Consortium (ICC) [13, 14], and subsequently incorporated into the National Comprehensive Cancer Network (NCCN) guidelines (S1 Table). Until accurate risk assessment and surveillance recommendations are specifically established for BRRS, such patients with pathogenic germline *PTEN* mutations undergo identical surveillance regimens as CS-PHTS. The identification and characterization of cancer-predisposing genes, particularly high-risk and high-penetrance genes, have facilitated accurate risk assessment, predictive testing for family members, and gene-specific tailored clinical management, helping in early cancer detection or prevention. However, this is only pertinent for known gene mutation positive patients.

Prospective community-based accrual of >3000 probands over >12 years revealed that ~25% of CS patients who met the diagnostic criteria harbored germline pathogenic *PTEN* mutations [15]. Moreover, many patients show clinical features reminiscent of CS but do not meet the ICC/NCCN criteria, and are referred to as CS-like that show extensive phenotypic heterogeneity with only 5% having germline *PTEN* mutations [16]. Germline *PTEN* mutations have been found in up to 60% of BRRS patients, and large *PTEN* deletions in ~10% [16–20]. Relevant to clinical practice, both CS and BRRS represent disorders with extensive degrees of phenotypic variability. CS is a clinical mimic with individual cancers observed in the general population or in other syndromes. Hence, even with subsequent identification of germline alterations in *SDHB-D* [21], *PIK3CA*/*AKT1* [22], *KLLN* [23], and *SEC23B* [24] in a subset of *PTEN*-wildtype CS patients in the research setting, ~50% remain wildtype for these known CS predisposition genes.

Although it is clear that PHTS patients are at an increased risk of developing certain cancers, the frequency of germline variants in known cancer susceptibility genes (besides *PTEN*) or in genes known to be associated with PHTS component cancers is unknown within this patient group. Currently, individuals with CS/CS-like and BRRS, even without variants in the known genes, are clinically managed as though they have *PTEN* variants. The overall objective of our study was to determine if known cancer predisposition genes or genes associated with other known heritable cancer syndromes are germane in CS/CS-like and BRRS. Furthermore, we attempt to associate clinical features with each candidate gene. If our hypothesis is correct, then our observations would suggest cancer risk management beyond those for PHTS.

## Materials and methods

### Ethics statement

Cleveland Clinic IRB approved our study protocol number 8458-PTEN and approved the research related to this protocol. Written informed consents were obtained from all research participants.

### Patient selection

Research participants were prospectively accrued broadly from cancer centers and the community (2011–2015). Eligible patients met at least the relaxed International Cowden Consortium (ICC) operational diagnostic criteria (S1 Table) [6]. Relaxed criteria are defined as full criteria minus one and such individuals are referred to as Cowden syndrome-like (CS-like) [14]. For all selected individuals, we reviewed the Cleveland Clinic (CC) score [15], a semi-quantitative score based on weighting clinical features and their ages of onset, and that estimates the pretest probability of finding a germline *PTEN* mutation (http://www.lerner.ccf.org/gmi/ccscore). Scoring criteria are evaluated and CC scores are derived by CPGH geneticists and genetic counselors based on medical records, or concurrently during physical exams. Between 2011 and 2014, we accrued a total of 1818 CS/CS-like/BRRS patients. For this exploratory analysis, we selected 92 patients based on clinical manifestations and phenotypic burden beyond threshold CC scores, or pathognomonic features such as Lhermitte-Duclos disease, and including all BRRS patients since it is a rare disease entity. These patients underwent *PTEN* mutation and deletion analyses as we have previously reported [10], and 5 were found to harbor germline pathogenic *PTEN* mutations and were hence excluded from further analysis. For the purposes of this analysis, we included the 87 *PTEN* wildtype patients for whole-exome sequencing (WES). Since all individuals are *PTEN*-wildtype, we used the CC score as a surrogate of phenotypic burden. We reviewed medical records, including pathology reports, for each research participant and extracted family history from clinical notes associated with cancer genetics and/or genetic counseling visits, where applicable and with the individuals’ consent.

### Gene curation

**Fig 1:**
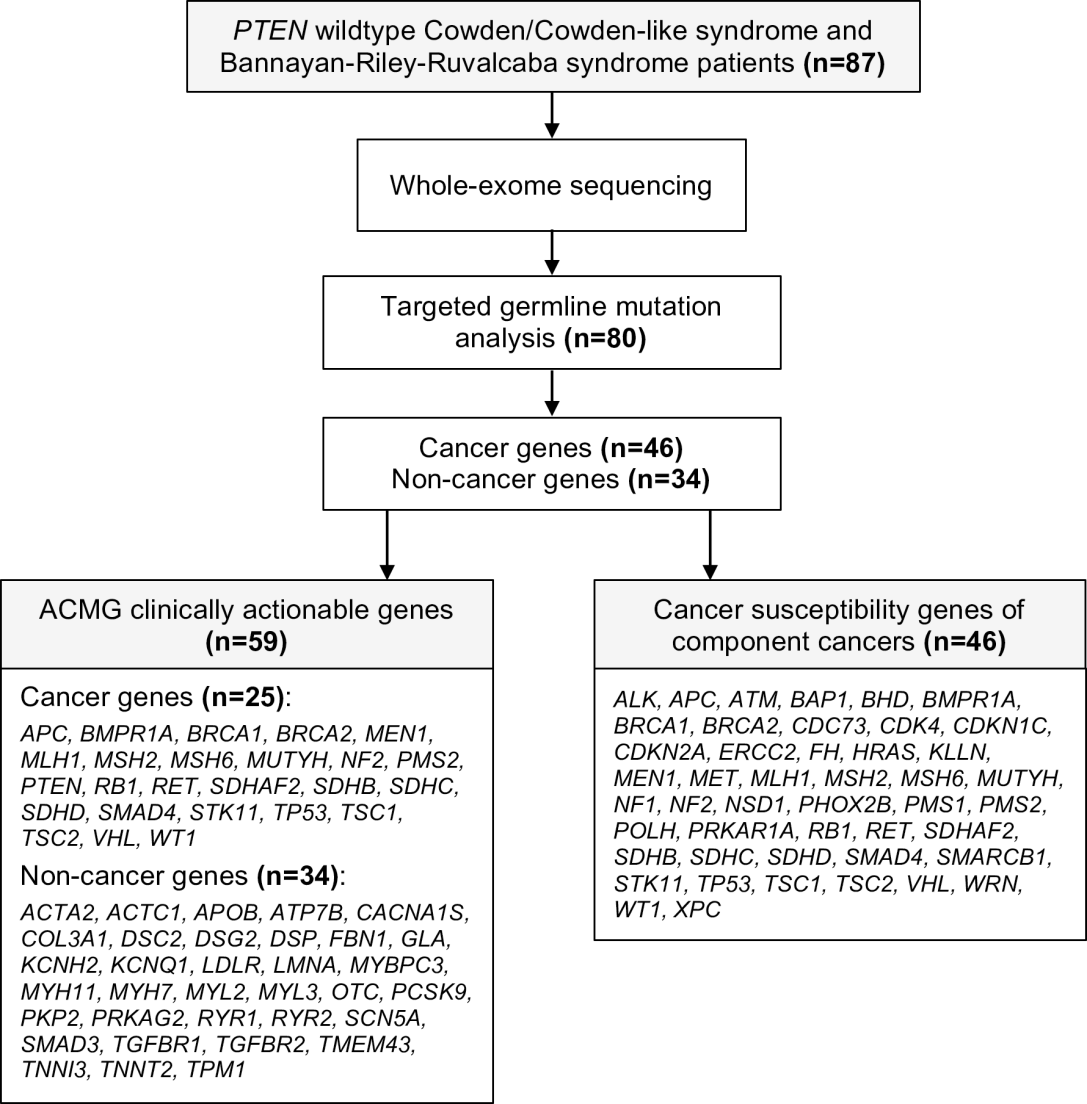
Study design and testing strategy. ACMG indicates American College of Medical Genetics and Genomics. Component cancers represent malignancies observed in PHTS patients.

We focused mutation analysis on 80 genes. These genes cover 59 genes included in the American College of Medical Genetics and Genomics (ACMG) guidelines, covering 25 candidate genes associated with inherited cancer syndromes and 34 non-cancer genes [25, 26]. We performed analysis of the non-cancer genes from the exome data as a negative control for non-random enrichment of phenotype-irrelevant germline genetic variants in patients with hereditary predisposition to cancer. We also curated 46 additional high penetrance genes known to harbor germline mutations in PHTS component cancers [27]. Excluding overlapping cancer genes from both datasets, the final analysis covered a total of 46 cancer-related genes, excluding *PTEN* (Fig 1).

### Next-generation sequencing and variant classification

Whole-exome sequencing (WES) was performed on germline genomic DNA extracted from peripheral-blood. Germline variant analysis was conducted based on Genome Analysis Toolkit (GATK, S1 Methods) [28]. We implemented the eXome Hidden Markov Model (XHMM) algorithm to call copy number variations (CNVs) from WES (S1 Methods, S2 Table).

WES variants were annotated and classified using Ingenuity Variant Analysis (IVA, Qiagen, Redwood City, California), which classifies variants according to ACMG guidelines [26, 29] (S1 Methods). We prioritized variants with a minor allele frequency (MAF) ≤0.01 (1%) as reported in 1000 Genomes (1000G), the National Heart, Lung, and Blood Institute Exome Sequencing Project (NHLBI-ESP6500), and the Exome Aggregation Consortium (ExAC) databases. All resultant variants were inspected through the Integrated Genomics Viewer (IGV) [30].

### The Cancer Genome Atlas (TCGA) data analysis

Approval for access to the TCGA germline DNA-sequence and clinical data was obtained from the Genomic Data Commons (GDC). We selected a total of 3476 cancer patients from 6 cancer types associated with PHTS (S3 Table). Germline variants were detected from blood-derived WES for 46 cancer-related gene genomic regions (S4 Table). We performed variant calling and classification with pipelines and criteria identical to the PHTS patient sequences. We retained all variants that passed the quality control filters. TCGA dataset was utilized as a representative of apparently sporadic component cancers to evaluate whether patients with hereditary predisposition to cancer would show relative enrichment of germline variants in the known studied cancer-related genes.

### Statistical analysis

Patient demographics were reported by age, sex, CC score, and clinical phenotypes. Student’s t test was used for significance testing. All statistical analyses were conducted using SPSS 24 software (SPSS Inc., Chicago, IL). Clinical characteristics and variant burden were compared using the Chi square test. All statistical tests were two-sided, and p values ≤0.05 were deemed significant.

## Results

### Patient characteristics

Eighty-seven eligible CS/CS-like and BRRS patients were identified based on wildtype *PTEN* mutation status and phenotypic burden (CC score ≥5). Females and males were equally distributed, with females accounting for 48.3% (n = 42) of patients (Table 1). The CC score ranged from 5 to 33 (median 15), indicating a high phenotype burden independent of *PTEN* mutation status. Overall, 43 (49.4%) patients had a reported cancer diagnosis, with 18 (41.9%) of those having second malignant primary neoplasms. In the 44 patients without cancer, the median age at consent was 23 years (range 1–65), compared to 49 years (range 15–72) in patients with at least one cancer diagnosis (p = 1.71x10^-8^). The former included 17 pediatric individuals (ages 1–14) and 9 adolescents and young adults (AYA, ages 16–29).

**Table 1:** Demographic and clinical characteristics of 87 *PTEN* wildtype Cowden/Cowden-like syndrome and Bannayan-Riley-Ruvalcaba syndrome patients.

Demographic and Clinical Characteristics		Number [Range]
***Median age at consent***		42 [1–72]
***Sex***		
**Female**	42	
**Male**	45	
***Median CC score***		15 [5–33]
***Thyroid cancer***	18	
**Follicular**	2	
**Papillary**	5	
**Follicular variant papillary**	10	
**Not otherwise specified (NOS)**	1	
***Benign thyroid***		
**Goiter**	33	
**Hashimoto’s thyroiditis**	9	
***Breast cancer***		
**Primary invasive**	14	
**Ductal**	6	
**Lobular**	1	
**Mixed**	1	
**Mucinous (colloid)**	1	
**NOS**	5	
**Carcinoma *in situ***	9	
***Benign breast***	26	
**Atypical ductal hyperplasia**	1	
**Atypical lobular hyperplasia**	1	
**Fibrocystic breast disease**	22	
**Breast papilloma**	2	
**Breast fibroadenoma**	7	
***Female genitourinary cancer***		
**Uterine cancer**	5	
**Endometrioid**	2	
**Choriocarcinoma**	1	
**NOS**	2	
***Renal cell cancer***	9	
**Clear cell**	1	
**Papillary**	4	
**Chromophobe**	2	
**Oncocytoma**	1	
**NOS**	1	
***Other CS/CS-like/BRRS features***		
**Macrocephaly**	64	
**Lhermitte-Duclos Disease**	8	
**Trichilemmoma**	12	
**Acral keratosis**	8	
**Papillomatous papules**	21	
**Lipoma / Fibroma / Hemangioma**	61	
**Melanoma**	2	
**GI polyps**	26	
**GI cancer**	5	
**Uterine fibroids**	14	
**Penile freckling**	31	

### Germline variants and copy number variation (CNVs) in *PTEN-*wildtype patients

From variant analysis of 46 cancer susceptibility genes, we identified 7 pathogenic and likely pathogenic germline variants in 7 genes (Table 2) in 7 (8%) of the 87 patients, namely, *MUTYH*, *RET*, *TSC2*, *BRCA1*, *BRCA2*, *ERCC2* and *HRAS* (Table 3). Irrespective of clinical cancer phenotype, the median age at consent of variant carriers was 47 years (range 5–71) with a median CC score of 14 (range 6–19). Of these patients, 5 (71.4%; median age at consent 58, range 26–71) had a reported cancer diagnosis, with 4 (80%) having more than one primary cancer. The median age at cancer diagnosis was 49 years (range 25–64, considering the earliest age at diagnosis when multiple primary cancers exist). The 2 remaining patients without cancer belong to the pediatric and AYA patient group (ages 5 and 8 years).

**Table 2:** Germline variants and copy number variation (CNVs) identified and associated syndromes.

Gene Variant	Classification	Associated Cancer or Syndrome (OMIM)	Patients with Variants (%)	dbSNP ID	1000G MAF	ExAC MAF	NHLBI ESP MAF	HGMD
***MUTYH***	Likely Pathogenic	Colon cancer (604933)	1 (1.15)	rs564930066	0.00040	0.00011	0	NA
NM_001128425 c.397G>C, p.D134H								
***RET***	Pathogenic	Multiple endocrine neoplasia type 2 (171400)	1 (1.15)	rs146646971	0	0.00002	0.00046	CM101836 (DM)
NM_020630 c.1998G>T, p.K666N		Familial medullary thyroid cancer (155240)						
***TSC2***	Pathogenic	Tuberous sclerosis complex (191100)	1 (1.15)	NA	0	0	0	NA
NM_000548 c.139-2A>G								
***BRCA1***	Pathogenic	Hereditary breast-ovarian cancer syndrome (604370)	1 (1.15)	rs80357711	0	0.00004	0	CD961844 (DM)
NM_007294 c.788-464delA, p.E1346fs*20								
***BRCA2***	Pathogenic	Hereditary breast-ovarian cancer syndrome (604370)	1 (1.15)	NA	0	0	0	CD011121 (DM)
NM_000059 c.4876_4877delAA, p.N1626fs*12								
***ERCC2***	Pathogenic	Xeroderma pigmentosum (278730) Trichothiodystrophy (601675)	1 (1.15)	rs587778271	0	0.00009	0.00008	CD013475 (DM)
NM_000400 c.1703_1704delTT, p.F568fs*2								
***HRAS***	NA^a^	Costello syndrome (218040)	1 (1.15)	NA	0	0	0	NA
chr11:533276–534375 duplication								

^a^CNVs were not classified by Ingenuity Variant Analysis (IVA)

Abbreviations: OMIM, Online Mendelian Inheritance in Man; dbSNP, Single Nucleotide Polymorphism database; 1000G, 1000 Genome Project; MAF, Minor Allele Frequency; ExAC, Exome Aggregation Consortium; NHLBI-ESP, National Heart, Lung, and Blood Institute Exome Sequencing Project; HGMD, Human Gene Mutation Database; DM, Disease-causing mutation; NA, Not Available

**Table 3:** Clinicopathological characteristics of patients with identified germline alterations.

Patient ID	CCF02255 *ERCC2*	CCF07060 *TSC2*	CCF08402 *MUTYH*	CCF07575 *RET*	CCF03206 *BRCA1*	CCF08133 *BRCA2*	CCF04432 *HRAS*
Age at Consent	47	26	8	58	64	71	5
Gender	F	F	M	M	F	M	M
CC Score	16	17	6	12	14	19	13
Macrocephaly							
Developmental Delay / Autism							
Breast Cancer	34				56,63		
Thyroid Cancer				55		68	
Endometrial Cancer					64		
Renal Cancer		26		56		64	
Colon Cancer		25					
Paraganglioma / Pheochromocytoma				49			
GI Polyps							
Benign Breast							
Uterine Fibroids / Ovarian Cysts							
Benign Nodules or Goiter							
Hashimoto’s Thyroiditis							
Trichilemmoma							
Acral Keratoses							
Papillomatous Papules							
Penile Freckling							
Lipoma							
Hemangioma							
Fibroma							
Mucosal pigmentation / Skin Cancer							
Related Clinical Presentation	N	Y	N	Y	Y	Y	Y
Family History of Cancer	N	Y	N	Y	N	Y	N

Variant positive patients with clinical features present in adult (light teal) and pediatric and adolescent and young adult (AYA) category (dark teal) of patients. Known age at diagnosis is shown as numbers in years within corresponding cells. Related clinical presentation is defined as known clinical features associated with the identified mutated genes. Family history of cancer is defined as the presence of first degree and/or second degree relatives with a reported cancer diagnosis. Y, yes; N, no.

We next looked at whether the identified genes were associated with their classic clinical phenotypes, hence indicating concordant gene-phenotype associations. We found such concordant gene-phenotype associations in 5 (71.4%) patients (Table 3). CCF07060, a 26-year old female, harbored *TSC2* c.139-2A>G splicing variant. Clinical MRI and post-op pathology reports indicated that this patient had clear cell renal cell carcinoma at age 26, noting that TSC is characterized by an increased risk of kidney cancer. We identified *RET* c.1998G>T, p.K666N variant in a 58-year old male with clinical features of CS (CCF07575). Gain-of-function germline heterozygous *RET* mutations cause multiple endocrine neoplasia type 2 (MEN 2), characterized by medullary thyroid carcinoma and pheochromocytoma. In addition to classic CS clinical features (macrocephaly, lipoma, renal cancer, thyroid cancer, goiter and Hashimoto’s thyroiditis), CCF07575 was also diagnosed with pheochromocytoma. Additionally, *BRCA1* c.788-464delA, p.E1346fs*20 truncating variant was identified in CCF03206, a 64-year old female with CS. This patient had bilateral invasive ductal carcinoma diagnosed in the right breast at age 57 and in the left breast at age 64, in addition to comedocarcinoma of the latter. Relatedly, CCF08133, a 71-year old male with *BRCA2* c.4876_4877delAA, p.N1626fs*12 truncating variant, had benign breast disease, not otherwise specified. Finally, an *HRAS* duplication was identified and functionally validated in a 5-year old male with global developmental delay (S1 Fig, S5 Table). Germline gain-of-function *HRAS* duplications have been reported in Costello syndrome [31], with developmental delay/intellectual disability as overlapping clinical features.

We found a family history of any cancer in 3 of the 7 (42.9%) patients with identified variants. Family history of any cancer is defined as the presence of first degree and/or second degree relatives with a reported cancer diagnosis, with the majority of pedigrees revealing a family history of PHTS-related cancers. In the remaining 80 (of the 87) patients without identified variants in the 46 cancer genes, 73 patients had available family history records. Of those 73, a family history of any cancer was found in 56 (76.7%), which was not significantly different than the subset with identified variants (OR = 0.233, 95% CI 0.0399–1.231, p = 0.085). Other secondary findings as per ACMG guidelines [26] included 3 non-cancer gene variants in 4 (4.6%) patients (S6 Table). We did not identify pathogenic or likely pathogenic variants in genes previously described in CS/CS-like patients, namely *SDHB*, *SDHD*, *PIK3CA*, *AKT1*, *SEC23B*, and *USF3* [21, 22, 24, 32].

### Germline variants identified in TCGA datasets

**Fig 2:**
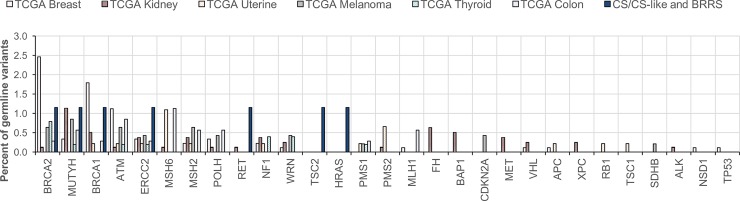
Comparison of pathogenic and likely pathogenic germline variant spectra and frequencies in CS/CS-like/BRRS patients and TCGA apparently sporadic component cancer patients. Solid-filled bars represent identified variant positive CS/CS-like and BRRS patients. Striped bars represent TCGA cancer patients with different colors indicating different cancer types. TCGA cancer types were selected because each is a PHTS component cancer. Abbreviations: CS, Cowden syndrome; BRRS, Bannayan-Riley-Ruvalcaba syndrome; OR, odds ratio; CI, confidence interval.

We analyzed TCGA datasets for germline variants in the 46 cancer susceptibility genes in 6 cancer types (n = 3476) that are observed in CS and BRRS, namely those of the breast (BRCA, n = 893), thyroid (THCA, n = 505), kidney (KIRC, n = 506; KIRP, n = 289), uterus (UCEC, n = 401; UCS, n = 57), colon (COAD, n = 355), and skin melanoma (SKCM, n = 470). We identified a total of 141 pathogenic and likely pathogenic germline heterozygous variants in 27 genes (Fig 2, S2 Fig, S7 Table). The most commonly mutated cancer susceptibility genes were *BRCA2* (n = 31), *BRCA1* (n = 22), *ATM* (n = 19), and *MUTYH* (n = 19). Other genes that showed ≥10 patients with germline variants included *ERCC2* (n = 11), *MSH2* (n = 11), and *MSH6* (n = 10).

The frequencies and spectra of germline variants in TCGA are compared to those found in our patient series (Fig 2). The number of patients with pathogenic and likely pathogenic germline variants (excluding CNVs) in the 46 cancer susceptibility genes (excluding *PTEN*) was higher in our patient series (n = 6/87 or 6.9%) compared to the TCGA series of apparently sporadic PHTS component cancers (n = 179/3476 or 5.1%), but this observation was not statistically significant (p = 0.46). However, even though the variant frequency is comparable between both datasets, we observed notable differences in the mutation spectrum. Although *BRCA1*, *BRCA2*, and *MUTYH* variants were observed in both patient series, we did not observe many of the TCGA-enriched mutated genes (eg. *ATM*, *MSH2*, *MSH6*) in CS/CS-like/BRRS patients. Conversely, we identified germline alterations in *TSC2* and *HRAS* in two of our patients, with no pathogenic or likely pathogenic germline variants in those genes identified in the PHTS component cancers within TCGA.

### Variants of uncertain significance (VUS)

We identified 64 VUS in the 46 cancer genes, occurring in 48 of 87 CS/CS-like and BRRS patients (55.2%). The genes most likely to have a VUS included *MET* (n = 11 [12.6%]), *BRCA2* (n = 7 [8.0%]), and *ALK* (n = 7 [8.0%]). In contrast, the TCGA component cancers yielded 998 VUS in 1222 out of 3476 patients (35.2%, p = 0.0002). The genes most likely to have a VUS included *MET* (n = 170 [10.6%]), *APC* (n = 167 [10.4%]), *ATM* (n = 140 [8.7%]), and *PMS1* (n = 116 [7.2%]) [S8 Table, S9 Table].

## Discussion

In the clinic, when PHTS is suspected, clinical genetic testing for *PTEN* is indicated. While a positive mutation result serves as molecular diagnostic, the implications of a negative result are less clear and patients are managed following guidelines established for those with *PTEN* mutations. CS/CS-like and BRRS are good models for hereditary cancer syndromes, where *PTEN*-informed clinical risk assessment results in gene-informed surveillance recommendations and clinical management of *PTEN* mutation positive patients [6]. However, despite all efforts towards seeking the genetic etiology for *PTEN* mutation negative CS/CS-like and BRRS, there remain half with “missing heritability.” Many CS and CS-like individuals evaluated in clinic have high CC scores [15] and no underlying germline *PTEN* mutations or large deletions, indicating high phenotypic burden independent of *PTEN* and a high likelihood that other susceptibility genes exist. CS is a great clinical mimic associated with multiple solid tumor types [6], with each of the latter component cancers associated with different predisposition genes. Here, we focused our study on ACMG-recommended genes and high penetrance genes known to be associated with PHTS component cancers because they have well-established cancer risks and clinical management guidelines that could lead to gene-specific risk assessments and management, instead of merely following PHTS guidelines in the absence of *PTEN* mutations.

We hypothesized that because our patient series represents individuals with elevated susceptibility for cancer, we will find an increased burden of variants in relevant known cancer-related genes. However, our data show that the prevalence of deleterious variants in multiple cancer-associated genes in our patient series was not notably higher than TCGA apparently sporadic cancer patients. This observation corroborates the complexity of hereditary cancer syndromes, and the likelihood that other non-classic cancer-associated genes may contribute to carcinogenesis in these patients. Indeed, we have recently identified *SEC23B* and *USF3* as candidate cancer susceptibility genes in *PTEN*-wildtype CS, and *TTN* in *PTEN*-wildtype BRRS [24, 32, 33]. Importantly, none of these genes were classically known to be associated with a cancer phenotype, reflecting that patients who remain with missing heritability could harbor germline variants in such non-classic genes. This certainly warrants further investigation, with careful phenotyping, variant segregation, and functional studies serving as strong foundations for such gene discovery studies. Moreover, the lack of enrichment of germline variants in hereditary cancers may also be explained by sample size differences and the constitution of patients between TCGA and our patient series. The latter is particularly true for some of our BRRS patients who belong to the pediatric, adolescent and young adult (AYA) patient category who may not have developed cancer yet, and which is virtually missing from TCGA cancers we investigated. Additionally, although we found germline variants in certain genes in both patient datasets (eg. *BRCA1*, *BRCA2*, and *MUTYH*), some genes were exclusively mutated in either CS/CS-like/BRRS patients or in TCGA PHTS component cancers. This observation indicates that perhaps rather than overall germline variant prevalence, the spectrum of mutated genes may better explain our patients with hereditary cancer versus patients with apparently sporadic cancer. In other words, the uniquely mutated genes and associated molecular signaling pathways involved may hint at the underlying mechanisms causing different diseases.

Close case review and clinical phenotypic analyses showed 5 of the 7 (71.4%) variant carrying CS/CS-like/BRRS patients had gene-relevant clinical presentations, while 3 (42.9%) had a family history of any type of cancer. The latter was comparable to individuals without identified germline variants. Intriguingly, we also found significant enrichment of VUS in these 46 cancer susceptibility genes in our hereditary cancer patient series compared to TCGA apparently sporadic cancer patients (55.2% versus 35.2%; OR = 2.3, 95% CI 1.5–3.5, p = 0.0002). We speculate that our patients could carry an increased burden of VUS, likely not only limited to these 46 genes but also throughout the genome, especially in genes relevant to pertinent molecular signaling pathways. With sufficient/accumulating prospective data that could lead to the reclassification of these variants, we suspect that the frequency of deleterious cancer-related gene variants is likely underrepresented in our patient series. One example is the enrichment of *MET* germline VUS in our patient series (n = 11 [12.6%]). Gain-of-function germline *MET* variants are classically associated with familial papillary renal cell carcinoma. *MET* p.T992I, found in 6 of our patients, has also been reported in familial colorectal [34] and differentiated thyroid cancers [35], both PHTS component malignancies, although the transforming capacity of this variant has been debated [36–38]. Only one patient with *MET* c.2908C>T, p.R970C (CCF07060) manifested with clear cell papillary renal cell cancer at age 26. CCF07265 had renal angiomyolipoma, a benign kidney neoplasm, more associated with TSC. CCF08441 had incidental hearing loss, noting that non-syndromic sensorineural hearing loss has been associated with *MET* recessive mutations [39]. Four of the *MET* variants we observed have also been reported in TCGA apparently sporadic cancers (p.M362T, p.S572N, p.R988C, and p.T992I) but with a notably lower overall frequency compared to our patient series (10 of 87 or 11.5% in CS/CS-like/BRRS versus 99 of 3476 or 2.8% in TCGA; OR = 4.43, 95% CI 2.11–8.56, p = 0.00032). These *MET* variants also show significant enrichment in our patient series compared to population frequency (10/174 alleles or 5.7% versus 72/5008 or 1.4% in the 1000 Genomes database; OR = 4.18, 95% CI 2.01–8.00, p = 4.49x10^-4^ and 1602/119669 or 1.3% in the ExAC database including TCGA; OR = 4.49, 95% CI 2.24–8.21, p = 1.67x10^-4^). Although the exact implications of this observation are less clear, our findings suggest that VUS such as the *MET* germline variants we reported may indeed play a unique, as yet unclear, role in the context of CS-related hereditary cancer predisposition.

It was also interesting to identify individuals harboring *BRCA1* and *BRCA2* variants in our patient series (Table 3). These patients presented with classic CS phenotypes (study inclusion criteria), including breast cancer or benign breast phenotype as one clinical feature. Therefore, these patients would not have been referred for HBOC genetic testing as a primary indication, particularly with inconclusive family history of related cancers. Relatedly, we identified a patient (CCF02255) with *ERCC2* frameshift truncating germline variant and breast cancer. Interestingly, *ERCC2* variants and mutations have been associated with breast cancer risk or clinical responses in specific contexts [40–42]. In fact, ERCC2 is a subunit of the TFIIH transcription/repair factor, a DNA helicase with an important function in nucleotide excision repair [43]. As such, autosomal recessive *ERCC2* germline mutations cause xeroderma pigmentosum (OMIM 278730) or trichothiodystrophy (OMIM 601675), both characterized by increased sensitivity to ultraviolet (UV) radiation and other skin abnormalities. Incidentally, our patient had clinically reported dermatitis, which may be induced due to photosensitivity. The patient also had a reported family history of GI polyps and colon cancer, the latter relevant to DNA damage responses and genome instability.

We also note the presence of multiple primary neoplasms in 4 of the 7 (57%) patients with identified germline variants. We find this intriguing as we have previously shown that 40% of individuals with germline pathogenic *PTEN* mutations have second malignant neoplasms (SMN) [10]. The risk of SMNs was 7-fold higher in *PTEN* mutation carriers compared to the US general population. In contrast, we note that of the 80 remaining patients without identified germline mutations in this study, only 14 (18%) had SMNs (OR = 6.101, 95% CI 1.143–35.98, p = 0.035). This observation, although based on a small sample size, suggests that individuals with SMNs are more likely to harbor germline variants in known cancer-related susceptibility genes. However, the absence of germline variants in the known cancer-related genes in the remaining wildtype syndromic patients with reported SMNs suggests the existence of germline alterations in other yet-to-be identified genes, and/or potential contribution of environmental factors such as prior exposure to chemotherapy or radiation therapy to SMN risk.

In this study, we intended to focus on the most well-defined heritable cancer disease genes listed by ACMG, with the addition of high-penetrance candidate genes for the hereditary cancer syndromes with CS component cancers [27]. One limitation of this study is the existence of a relatively large number of cancer-related candidate genes not covered in our current analysis, including those with burgeoning evidence of being associated with cancer. However, many of the latter genes remain low-to-moderate penetrance and clinically inactionable (e.g., *BRIP1*, *NBN*, *PALB2* missense variants). As proof-of-principle, expanding our gene list to include select PHTS component cancer-relevant low-to-moderate penetrance genes, genes recommended for testing by the National Comprehensive Cancer Network (NCCN), and other genes that are medically actionable with established management and risk-reduction guidelines (S10 Table) resulted in 20 additional genes that we have now re-evaluated. These include: *BLM*, *BRIP1*, *CDH1*, *CHEK2*, *DICER1*, *EPCAM*, *FLCN*, *GALNT12*, *GREM1*, *MAX*, *MITF*, *NBN*, *PALB2*, *POLD1*, *POLE*, *POT1*, *RAD51C*, *RAD51D*, *SDHA*, and *TMEM127*. We only found two additional moderate penetrance pathogenic/likely pathogenic *CHEK2* variants (p.I157T, rs17879961; p.R145W, rs137853007) in two additional patients (S11 Table). These data further support that the “missing heritability” in the remaining wildtype patients is unlikely to be due to the contribution of other known cancer-related genes, and the likely existence of non-classic cancer-associated genes or non-traditional mechanisms of tumorigenesis (e.g., epigenetics). The latter processes warrant further investigation.

A key strength of this study is the prospective accrual of an incident series of CS/CS-like and BRRS patients, reflecting clinical practice. *In toto*, our observations reveal that germline variants in a variety of known cancer genes could potentially explain a subset of *PTEN*-wildtype CS/CS-like and BRRS patients (8%). These known cancer genes are associated with their respective phenotypes, perhaps suggesting management beyond that for PHTS. Accordingly, from a clinical point of view, mutation-positive patients should be medically managed as per the identified genes, particularly when guidelines are available. However, other CS/BRRS syndrome-related features and component cancers cannot be ignored without further evidence and at this time, should be managed as per CS/BRRS guidelines. The remaining high proportion of CS/CS-like and BRRS patients beyond this series, with clear phenotypic burden and unidentified genetic etiology, also call for more comprehensive genomic coverage, extending mutation analysis beyond known genes and into relevant signaling pathways, and the possible existence of alternative epigenetic mechanisms or non-classic cancer-related genes, as we have previously reported [24, 32]. Our findings can be used to understand the utility of next-generation sequencing approaches, including broader cancer multigene panels, in high-risk patients with heritable cancer syndromes of unknown genetic etiology, irrespective of age, cancer phenotype, or family history. Our observations also underscore that cancer predisposition genes, associated with specific clinical features and management guidelines, beyond those linked to a named heritable cancer syndrome(s), here, CS/CS-like and BRRS, may be germane to clinical practice.

## Supporting information

S1 Methods Next-generation sequencing and variant calling and variant filtering and prioritization. (PDF)

S1 Table International Cowden Consortium (ICC) operational diagnostic criteria (Ver. 2006). Abbreviations: LDD, Lhermitte-Duclos Disease; CS, Cowden syndrome.
(PDF)

S2 Table TaqMan copy number assays to validate copy number variation (CNVs). Abbreviations: hg38, Genome Reference Consortium Human Build 38 reference genome assembly.
(PDF)

S3 Table CS/CS-like and BRRS component cancers examined in The Cancer Genome Atlas (TCGA). (PDF)

S4 Table Cancer gene genomic regions studied. Abbreviations: hg19/38, Human Build 19 and 38 reference genome assemblies.
(PDF)

S5 Table Germline CNVs identified in CS/CS-like and BRRS patients by XHMM. Abbreviations: CNV, copy number variants; DEL, deletion; DUP, duplication; Kb, kilobases; Q_SOME, Phred-scaled quality of CNV event in the interval.
(PDF)

S6 Table Germline variants identified in ACMG non-cancer genes in CS/CS-like and BRRS. ^a^Patients harboring these variants did not undergo related medical workup in our clinic, and pedigree did not indicate a family history of the associated syndromes. Abbreviations: OMIM, Online Mendelian Inheritance in Man; No., number.
(PDF)

S7 Table Germline variants identified in cancer genes in TCGA component cancers. Abbreviations: Chr., chromosome; dbSNP, Single Nucleotide Polymorphism database; 1000G, 1000 Genomes Project; NA, not available; HGMD, Human Gene Mutation Database; DM, disease-causing mutation; OMIM, Online Mendelian Inheritance in Man; BRCA, Breast Invasive Carcinoma; THCA, Thyroid Carcinoma; KIRC, Kidney Renal Clear Cell Carcinoma; KIRP, Kidney Renal Papillary Cell Carcinoma; UCEC, Uterine Corpus Endometrial Carcinoma; UCS, Uterine Carcinosarcoma; COAD, Colon Adenocarcinoma; SKCM, Skin Cutaneous Melanoma. *We identified 2 pathogenic germline *PTEN* heterozygous mutations in one breast cancer patient (c.388C>T, p.R130X) and one endometrial cancer patient (c.518G>A, p.R173H).
(XLSX)

S8 Table Germline VUS identified in cancer genes in CS/CS-like and BRRS. Abbreviations: Chr., chromosome; dbSNP, Single Nucleotide Polymorphism database; 1000G, 1000 Genomes Project; NA, not available; HGMD, Human Gene Mutation Database; DM, disease-causing mutation; OMIM, Online Mendelian Inheritance in Man.
(XLSX)

S9 Table Germline VUS identified in cancer genes in TCGA component cancers. Abbreviations: Chr., chromosome; dbSNP, Single Nucleotide Polymorphism database; 1000G, 1000 Genomes Project; NA, not available; HGMD, Human Gene Mutation Database; DM, disease-causing mutation; OMIM, Online Mendelian Inheritance in Man; BRCA, Breast Invasive Carcinoma; THCA, Thyroid Carcinoma; KIRC, Kidney Renal Clear Cell Carcinoma; KIRP, Kidney Renal Papillary Cell Carcinoma; UCEC, Uterine Corpus Endometrial Carcinoma; UCS, Uterine Carcinosarcoma; COAD, Colon Adenocarcinoma; SKCM, Skin Cutaneous Melanoma.
(XLSX)

S10 Table List of PHTS-relevant re-evaluated genes. ^a^Genes include select PHTS component cancer-relevant low-to-moderate penetrance genes, genes recommended for testing by the National Comprehensive Cancer Network (NCCN), and other genes that are medically actionable with established management and risk-reduction guidelines. Abbreviations: PCC, pheochromocytoma; PGL, paraganglioma
(PDF)

S11 Table Demographic and clinical characteristics of patients with identified *CHEK2* variants. Abbreviations: CCF ID, Cleveland Clinic identification number; CC score, Cleveland Clinic score; GI, gastrointestinal.
(PDF)

S1 Fig Validation of copy number variation (CNV) identified in patient CCF04432. A. XHMM output showing duplication of *HRAS* (green line) in patient CCF04432 compared to the other patient exomes included in the analysis (gray lines). B. Quantitative PCR analysis of the *HRAS* locus using gene-specific TaqMan probes. Actin was used as an internal housekeeping gene for normalization.
(TIF)

S2 Fig Spectrum of germline cancer gene variants identified in TCGA component cancers. Abbreviations: BRCA, Breast Invasive Carcinoma; THCA, Thyroid Carcinoma; KIRC, Kidney Renal Clear Cell Carcinoma; KIRP, Kidney Renal Papillary Cell Carcinoma; UCEC, Uterine Corpus Endometrial Carcinoma; UCS, Uterine Carcinosarcoma; COAD, Colon Adenocarcinoma; SKCM, Skin Cutaneous Melanoma.
(TIF)
