# Supplementary material for: Unexpected cancer-predisposition gene variants in Cowden syndrome and Bannayan-Riley-Ruvalcaba syndrome patients without underlying germline PTEN mutations
Source: PLoS Genet. 2018 Apr 23;14(4):e1007352. doi: 10.1371/journal.pgen.1007352 (PMC5933810; doi:10.1371/journal.pgen.1007352)
Supplement: S1 Methods — (PDF) [file pgen.1007352.s001.pdf]

Next-Generation Sequencing, Variant Calling and Validation

WES was performed on germline genomic DNA extracted from peripheral-blood leukocytes of eligible patients. Exome enrichment was performed with the TruSeq SBS v.3 or Nextera Rapid Capture Exome (Illumina), and subsequent 100 bp paired-end sequencing was performed with Illumina HiSeq 2000 or 2500 platforms. Raw sequencing reads were mapped to the hg19 human reference haploid genome sequence using the Burrows-Wheeler Aligner (BWA version 0.6.1) [1]. Indel realignment, base and quality score recalibrations, and removal of PCR duplicates from the resultant Binary Alignment Map (BAM) files were performed using the Genome Analysis Toolkit (GATK) [2, 3], Sequence Alignment/Map (SAMtools) and Picard [4]. Variant discovery and genotype calling of single nucleotide variations (SNVs) and short insertions and deletions (indels, <50 bp) were performed using the GATK Haplotype Caller. Prioritized gene variants were validated by Sanger sequencing. Resultant chromatograms were analyzed with Mutation Surveyor DNA Variant Analysis Software (SoftGenetics).

We implemented the eXome Hidden Markov Model (XHMM) algorithm to call copy number variations (CNVs) from WES, using default parameters. XHMM makes use of principal component analysis to normalize sequencing read depth and a hidden Markov model, while providing corresponding quality metrics for downstream prioritisation [5, 6]. We extracted data corresponding to the 46 cancer susceptibility genes under study. The mean per-target depth of coverage across all samples was 50. An XHMM quality score (SQ or Q_some) of 60 was used as a cut-off threshold for CNV filtration and prioritization. Prioritized CNVs were validated using pre-designed TaqMan Copy Number assays (S1 Fig, S2 Table).

Variant Filtering and Prioritization

WES variants were annotated using Ingenuity Variant Analysis (IVA, Qiagen, Redwood City, California), last accessed in August 2017. Filtering settings for ACMG pathogenicity classifications

included base call quality of at least 20, read depth of at least 10, genotype quality score of at least 30, and existence outside the top 5% most exonically variable 100 base windows in healthy public genomes (1000 Genomes Project, 1000G, August 2015 release). We extracted targeted gene regions corresponding to the 46 cancer susceptibility genes of interest. We prioritized variants with a minor allele frequency (MAF) ≤ 0.01 (1%) as reported in 1000G or the National Heart, Lung, and Blood Institute Exome Sequencing Project (NHLBI-ESP6500) databases. All resultant variants were inspected through the Integrated Genomics Viewer (IGV) [7, 8].

References

1. Li H, Durbin R. Fast and accurate short read alignment with Burrows-Wheeler transform. Bioinformatics. 2009;25(14):1754-60. Epub 2009/05/20. doi: 10.1093/bioinformatics/btp324. PubMed PMID: 19451168; PubMed Central PMCID: PMC2705234.

2. McKenna A, Hanna M, Banks E, Sivachenko A, Cibulskis K, Kernytsky A, et al. The Genome Analysis Toolkit: a MapReduce framework for analyzing next-generation DNA sequencing data. Genome research. 2010;20(9):1297-303. Epub 2010/07/21. doi: 10.1101/gr.107524.110. PubMed PMID: 20644199; PubMed Central PMCID: PMC2928508.

3. DePristo MA, Banks E, Poplin R, Garimella KV, Maguire JR, Hartl C, et al. A framework for variation discovery and genotyping using next-generation DNA sequencing data. Nature genetics. 2011;43(5):491-8. Epub 2011/04/12. doi: 10.1038/ng.806. PubMed PMID: 21478889; PubMed Central PMCID: PMC3083463.

4. Li H, Handsaker B, Wysoker A, Fennell T, Ruan J, Homer N, et al. The Sequence Alignment/Map format and SAMtools. Bioinformatics. 2009;25(16):2078-9. Epub 2009/06/10. doi: 10.1093/bioinformatics/btp352. PubMed PMID: 19505943; PubMed Central PMCID: PMC2723002.

5. Fromer M, Moran JL, Chambert K, Banks E, Bergen SE, Ruderfer DM, et al. Discovery and statistical genotyping of copy-number variation from whole-exome sequencing depth. American

journal of human genetics. 2012;91(4):597-607. doi: 10.1016/j.ajhg.2012.08.005. PubMed PMID: 23040492; PubMed Central PMCID: PMC3484655.

6. Poultney CS, Goldberg AP, Drapeau E, Kou Y, Harony-Nicolas H, Kajiwara Y, et al. Identification of small exonic CNV from whole-exome sequence data and application to autism spectrum disorder. American journal of human genetics. 2013;93(4):607-19. doi: 10.1016/j.ajhg.2013.09.001. PubMed PMID: 24094742; PubMed Central PMCID: PMC3791269.

7. Robinson JT, Thorvaldsdottir H, Winckler W, Guttman M, Lander ES, Getz G, et al. Integrative genomics viewer. Nat Biotechnol. 2011;29(1):24-6. Epub 2011/01/12. doi: 10.1038/nbt.1754. PubMed PMID: 21221095; PubMed Central PMCID: PMC3346182.

8. Thorvaldsdottir H, Robinson JT, Mesirov JP. Integrative Genomics Viewer (IGV): high-performance genomics data visualization and exploration. Brief Bioinform. 2013;14(2):178-92. Epub 2012/04/21. doi: 10.1093/bib/bbs017. PubMed PMID: 22517427; PubMed Central PMCID: PMC3603213.
