# Supplementary material for: Unexpected cancer-predisposition gene variants in Cowden syndrome and Bannayan-Riley-Ruvalcaba syndrome patients without underlying germline PTEN mutations
Source: PLoS Genet. 2018 Apr 23;14(4):e1007352. doi: 10.1371/journal.pgen.1007352 (PMC5933810; doi:10.1371/journal.pgen.1007352)
Supplement: S1 Table — Abbreviations: LDD, Lhermitte-Duclos Disease; CS, Cowden syndrome. (PDF) [file pgen.1007352.s002.pdf]

<table>
<tr>
<td>Pathognomonic
Adult Lhermitte-Duclos disease
Mucocutaneous lesions
Trichilemmomas, facial
Acral keratoses
Papillomatous papules
Mucosal lesions</td>
<td>Major
Breast cancer
Thyroid cancer (nonmedullary)
Macrocephaly (i.e., ≥ 97th percentile)
Endometrial cancer</td>
<td>Minor
Other thyroid lesions (eg, adenoma, multinodular goiter)
Mental retardation (i.e., IQ ≤ 75)
GI hamartomas
Fibrocystic breast disease
Lipomas
Fibromas
Genitourinary tumors (especially renal cell carcinoma)
Genitourinary malformations
Uterine fibroids</td>
</tr>
<tr>
<td colspan="3">Operational diagnosis in an individual
Any of following:
Mucocutaneous lesions alone, if ≥ six facial papules (three of which must be trichilemmomas)
Cutaneous facial papules and oral mucosal papillomatosis
Oral mucosal papillomatosis and acral keratoses
≥ Six palmoplantar keratoses
≥ Two major criteria (one of which must be macrocephaly or LDD)
One major and ≥ three minor criteria
≥ Four minor criteria</td>
</tr>
<tr>
<td colspan="3">Operational diagnosis in a family where one individual is diagnostic for CS
Any one pathognomonic criterion
Any one major criteria ± minor criteria
Two minor criteria
History of Bannayan-Riley-Ruvalcaba syndrome</td>
</tr>
</table>
