# Supplementary material for: Unexpected cancer-predisposition gene variants in Cowden syndrome and Bannayan-Riley-Ruvalcaba syndrome patients without underlying germline PTEN mutations
Source: PLoS Genet. 2018 Apr 23;14(4):e1007352. doi: 10.1371/journal.pgen.1007352 (PMC5933810; doi:10.1371/journal.pgen.1007352)
Supplement: S2 Table — Abbreviations: hg38, Genome Reference Consortium Human Build 38 reference genome assembly. (PDF) [file pgen.1007352.s003.pdf]

Gene	Taqman Probe ID	Taqman Probe Description on hg38
BRCA2	Hs01546666_cn	Within exon 11
BRCA2	Hs05297705_cn	Within intron 11
NF2	Hs00812496_cn	Overlaps Intron 9 - Exon 10
NF2	Hs00469182_cn	Overlaps Intron 14 - Exon 15
HRAS	Hs00134726_cn	Overlaps Intron 3 - Exon 3
HRAS	Hs00029675_cn	Within Exon 4
TSC1	Hs02507977_cn	Overlaps Intron 9 - Exon 10
TSC1	Hs01076986_cn	Overlaps Intron 16 - Exon 16
TSC1	Hs02744828_cn	Overlaps Intron 23 - Exon 23
MUTYH	Hs01327911_cn	Overlaps Intron 10 - Intron 11
MUTYH	Hs01869574_cn	Overlaps Intron 16 - Intron 17
