# Supplementary material for: Unexpected cancer-predisposition gene variants in Cowden syndrome and Bannayan-Riley-Ruvalcaba syndrome patients without underlying germline PTEN mutations
Source: PLoS Genet. 2018 Apr 23;14(4):e1007352. doi: 10.1371/journal.pgen.1007352 (PMC5933810; doi:10.1371/journal.pgen.1007352)
Supplement: S3 Table — (PDF) [file pgen.1007352.s004.pdf]

TCGA Study	Description	Number of Patients	Median Age (Range)
**BRCA**	Breast Invasive Carcinoma	893	57 (26-89)
**THCA**	Thyroid Carcinoma	505	46 (15-89)
**KIRC**	Kidney Renal Clear Cell Carcinoma	506	60 (26-89)
**KIRP**	Kidney Renal Papillary Cell Carcinoma	289	61 (28-87)
**UCEC**	Uterine Corpus Endometrial Carcinoma	401	61 (31-89)
**UCS**	Uterine Carcinosarcoma	57	68 (51-89)
**COAD**	Colon Adenocarcinoma	355	68 (31-89)
**SKCM**	Skin Cutaneous Melanoma	470	58 (15-89)
**Total**	TCGA CS/CS-like and BRRS component cancers	**3476**	**60 (15-89)**
