# Supplementary material for: Unexpected cancer-predisposition gene variants in Cowden syndrome and Bannayan-Riley-Ruvalcaba syndrome patients without underlying germline PTEN mutations
Source: PLoS Genet. 2018 Apr 23;14(4):e1007352. doi: 10.1371/journal.pgen.1007352 (PMC5933810; doi:10.1371/journal.pgen.1007352)
Supplement: S4 Table — Abbreviations: hg19/38, Human Build 19 and 38 reference genome assemblies. (PDF) [file pgen.1007352.s005.pdf]

No.	Gene Symbol	Chr.	Start (hg19)	End (hg19)	Start (hg38)	End (hg38)
1	*ALK*	2	29415639	30144477	29192773	29921566
2	*APC*	5	112073555	112181936	112737887	112846239
3	*ATM*	11	108093558	108239826	108222831	108369099
4	*BAP1*	3	52435019	52444121	52401012	52410350
5	*BMPR1A*	10	88516395	88684945	86755786	86927969
6	*BRCA1*	17	41196311	41277500	43044294	43125370
7	*BRCA2*	13	32889616	32973809	32315473	32400266
8	*CDC73*	1	193091087	193223942	193122016	193253901
9	*CDK4*	12	58141509	58146230	57747726	57752521
10	*CDKN1C*	11	2904447	2906995	2883212	2885881
11	*CDKN2A*	9	21967750	21975132	21968055	21974866
12	*ERCC2*	19	45854648	45873845	45349836	45370618
13	*FH*	1	241660856	241683085	241497602	241519761
14	*HRAS*	11	532241	535567	532242	535550
15	*KLLN*	10	89618917	89623194	86756649	86932838
16	*MEN1*	11	64570985	64578188	64803514	64811294
17	*MET*	7	116312458	116438440	116672391	116798386
18	*MLH1*	3	37034840	37092337	36993331	37050918
19	*MSH2*	2	47630205	47710367	47402968	47483228
20	*MSH6*	2	48010220	48034092	47783081	47810101
21	*MUTYH*	1	45794913	45806142	45329241	45340388
22	*NF1*	17	29421944	29704695	31094926	31377677
23	*NF2*	22	29999544	30094589	29603557	29698598
24	*NSD1*	5	176560832	176727214	177133924	177300215
25	*PHOX2B*	4	41746098	41750987	41744081	41748970
26	*PMS1*	2	190648810	190742355	189784084	189877628
27	*PMS2*	7	6012869	6048737	5973238	6009125
28	*POLH*	6	43543877	43588260	43576149	43615660
29	*PRKAR1A*	17	66508519	66529570	68512378	68533423
30	*PTEN*	10	89623194	89731687	87859160	87971930
31	*RB1*	13	48877882	49056026	48303774	48481986
32	*RET*	10	43572516	43622952	43077026	43130351
33	*SDHAF2*	11	61197596	61214239	61430125	61446767
34	*SDHB*	1	17345224	17380665	17018721	17054170
35	*SDHC*	1	161284165	161334535	185045363	185102608
36	*SDHD*	11	111957547	111966525	112086772	112095794
37	*SMAD4*	18	48556582	48611411	51029613	51085045
38	*SMARCB1*	22	24129117	24176705	23786962	23834516
39	*STK11*	19	1205797	1228434	1206463	1228435
40	*TP53*	17	7571719	7590868	7668401	7687538
41	*TSC1*	9	135766734	135820020	132891347	132944621
42	*TSC2*	16	2097895	2138721	2047464	2088712
43	*VHL*	3	10183318	10195354	10141007	10152220
44	*WRN*	8	30890777	31031277	31033800	31173769
45	*WT1*	11	32409321	32457081	32387774	32435630
46	*XPC*	3	14186647	14220172	14145146	14178783
