# Supplementary material for: Unexpected cancer-predisposition gene variants in Cowden syndrome and Bannayan-Riley-Ruvalcaba syndrome patients without underlying germline PTEN mutations
Source: PLoS Genet. 2018 Apr 23;14(4):e1007352. doi: 10.1371/journal.pgen.1007352 (PMC5933810; doi:10.1371/journal.pgen.1007352)
Supplement: S5 Table — Abbreviations: CNV, copy number variants; DEL, deletion; DUP, duplication; Kb, kilobases; Q_SOME, Phred-scaled quality of CNV event in the interval. (PDF) [file pgen.1007352.s006.pdf]

Sample	CNV	Chr.	Interval	Size (Kb)	Genes of Interest	Q_SOME
**CCF08178**	DEL	chr13	32893213-32930746	37.53	*BRCA2*	74
**CCF04432**	DEL	chr22	30057193-30077590	20.4	*NF2*	60
**CCF04432**	DUP	chr11	533276-534375	1.1	*HRAS*	80
**CCF04432**	DEL	chr9	135772570-135797360	24.79	*TSC1*	92
**CCF04432**	DEL	chr6	43565432-43578460	13.03	*POLH*	62
**CCF00344**	DEL	chr1	45797332-45798506	1.18	*MUTYH*	99
