# Supplementary material for: Unexpected cancer-predisposition gene variants in Cowden syndrome and Bannayan-Riley-Ruvalcaba syndrome patients without underlying germline PTEN mutations
Source: PLoS Genet. 2018 Apr 23;14(4):e1007352. doi: 10.1371/journal.pgen.1007352 (PMC5933810; doi:10.1371/journal.pgen.1007352)
Supplement: S6 Table — aPatients harboring these variants did not undergo related medical workup in our clinic, and pedigree did not indicate a family history of the associated syndromes. Abbreviations: OMIM, Online Mendelian Inheritance in Man; No., number. (PDF) [file pgen.1007352.s007.pdf]

| Gene Variant | Classification | Associated Syndrome (OMIM) | No. of Patients[a] with Variants (%) |
|---|---|---|---|
| ***DSC2***<br>NM_024422: c.2686_2687dupGA, p.A897fs*4 | Pathogenic | Arrhythmogenic right-ventricular cardiomyopathy (609040) | 2 (2.30) |
| ***GLA***<br>NM_000169: c.937G>T, p.D313Y | Pathogenic | Hypertrophic cardiomyopathy, dilated cardiomyopathy (301500) | 1 (1.15) |
| ***TGFBR2***<br>NM_001024847: c.1234G>A, p.V412M | Likely Pathogenic | Loeys-Dietz syndrome (610168) | 1 (1.15) |
