# Supplementary material for: Unexpected cancer-predisposition gene variants in Cowden syndrome and Bannayan-Riley-Ruvalcaba syndrome patients without underlying germline PTEN mutations
Source: PLoS Genet. 2018 Apr 23;14(4):e1007352. doi: 10.1371/journal.pgen.1007352 (PMC5933810; doi:10.1371/journal.pgen.1007352)
Supplement: S10 Table — aGenes include select PHTS component cancer-relevant low-to-moderate penetrance genes, genes recommended for testing by the National Comprehensive Cancer Network (NCCN), and other genes that are medically actionable with established management and risk-reduction guidelines. Abbreviations: PCC, pheochromocytoma; PGL, paraganglioma (PDF) [file pgen.1007352.s011.pdf]

Cancer type	Genes[a]
Breast	*ATM, BRCA1, BRCA2, CDH1, CHEK2, NBN, NF1, PALB2, PTEN, STK11, TP53*
Breast and gynaecological	*ATM, BRCA1, BRCA2, BRIP1, CDH1, CHEK2, EPCAM, MLH1, MSH2, MSH6, NBN, NF1, PALB2, PMS2, PTEN, RAD51C, RAD51D, STK11, TP53*
Thyroid	*APC, CHEK2, DICER1, PRKAR1A, PTEN, RET, TP53*
Kidney	*BAP1, EPCAM, FH, FLCN, MET, MITF, MLH1, MSH2, MSH6, PMS2, PTEN, SDHB, SDHC, SDHD, TP53, TSC1, TSC2, VHL*
Colon	*APC, BMPR1A, EPCAM, MLH1, MSH2, MSH6, MUTYH, PMS2, PTEN, SMAD4, STK11, TP53, BLM, CHEK2, GALNT12, GREM1, POLD1, POLE*
Melanoma	*BAP1, BRCA2, CDK4, CDKN2A, MITF, POT1, PTEN, RB1, TP53*
PCC/PGL	*MAX, NF1, RET, SDHA, SDHAF2, SDHB, SDHC, SDHD, TMEM127, VHL*
