# Supplementary material for: Unexpected cancer-predisposition gene variants in Cowden syndrome and Bannayan-Riley-Ruvalcaba syndrome patients without underlying germline PTEN mutations
Source: PLoS Genet. 2018 Apr 23;14(4):e1007352. doi: 10.1371/journal.pgen.1007352 (PMC5933810; doi:10.1371/journal.pgen.1007352)
Supplement: S11 Table — Abbreviations: CCF ID, Cleveland Clinic identification number; CC score, Cleveland Clinic score; GI, gastrointestinal. (PDF) [file pgen.1007352.s012.pdf]

**CCF ID**	***CHEK2* variant**	**Age at consent**	**Gender**	**CC score**	**Clinical features**
CCF04532	p.I157T (rs17879961)	37	M	17	Penile freckling, lipoma, GI polyps, rectal cancer (age 26)
CCF00449	p.R145W (rs137853007)	42	F	11	Follicular variant papillary thyroid cancer (age 41), goiter, fibrocystic breast disease, breast fibroadenoma and breast papilloma, lipoma, fibroma, GI polyps, GI cancer, uterine fibroids, genitourinary malformations
