# Supplementary figures and images for: Unexpected cancer-predisposition gene variants in Cowden syndrome and Bannayan-Riley-Ruvalcaba syndrome patients without underlying germline PTEN mutations
Source: PLoS Genet. 2018 Apr 23;14(4):e1007352. doi: 10.1371/journal.pgen.1007352 (PMC5933810; doi:10.1371/journal.pgen.1007352)

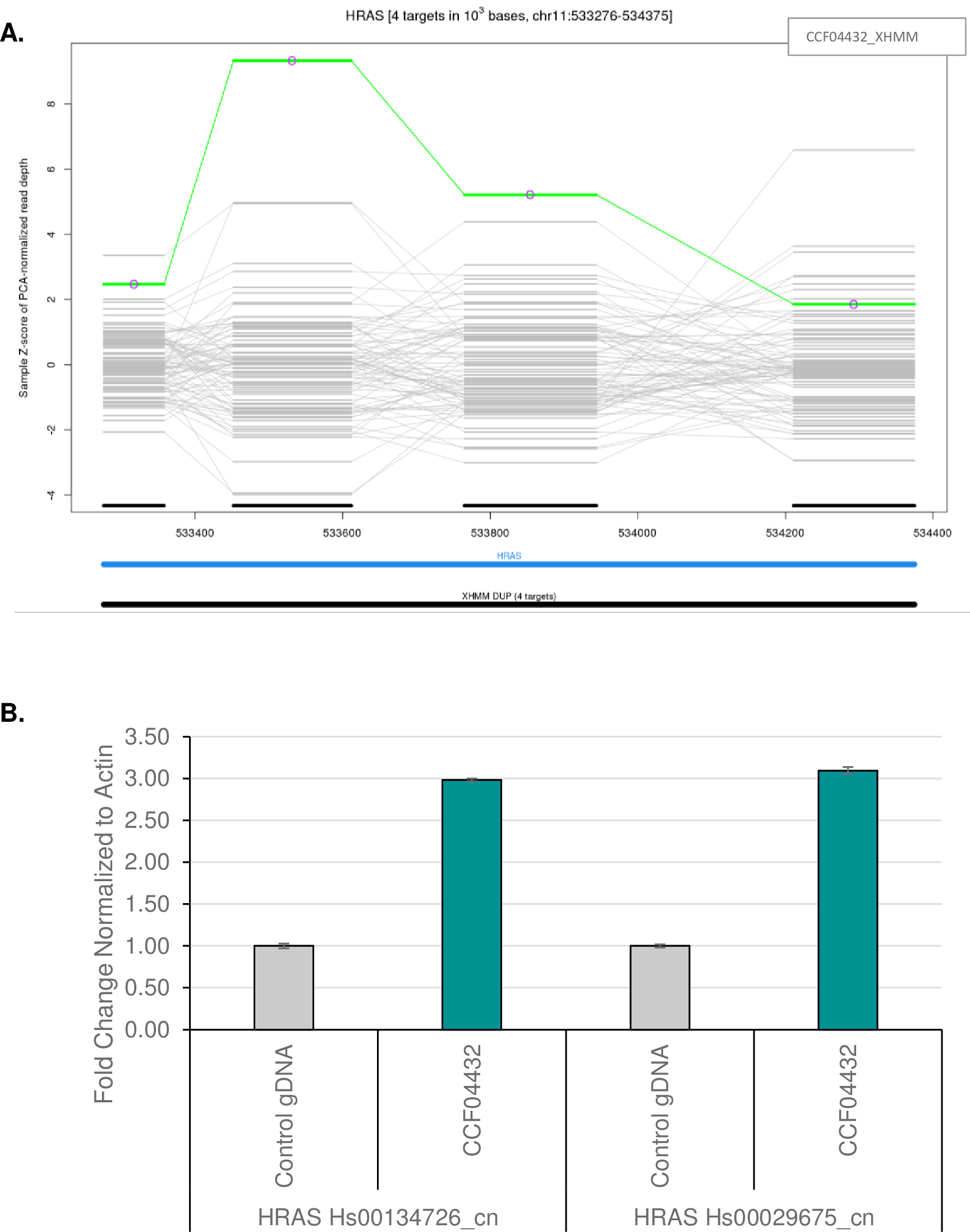

Supplement: S1 Fig — A. XHMM output showing duplication of HRAS (green line) in patient CCF04432 compared to the other patient exomes included in the analysis (gray lines). B. Quantitative PCR analysis of the HRAS locus using gene-specific TaqMan probes. Actin was used as an internal housekeeping gene for normalization. (TIF) [file pgen.1007352.s013.tif]

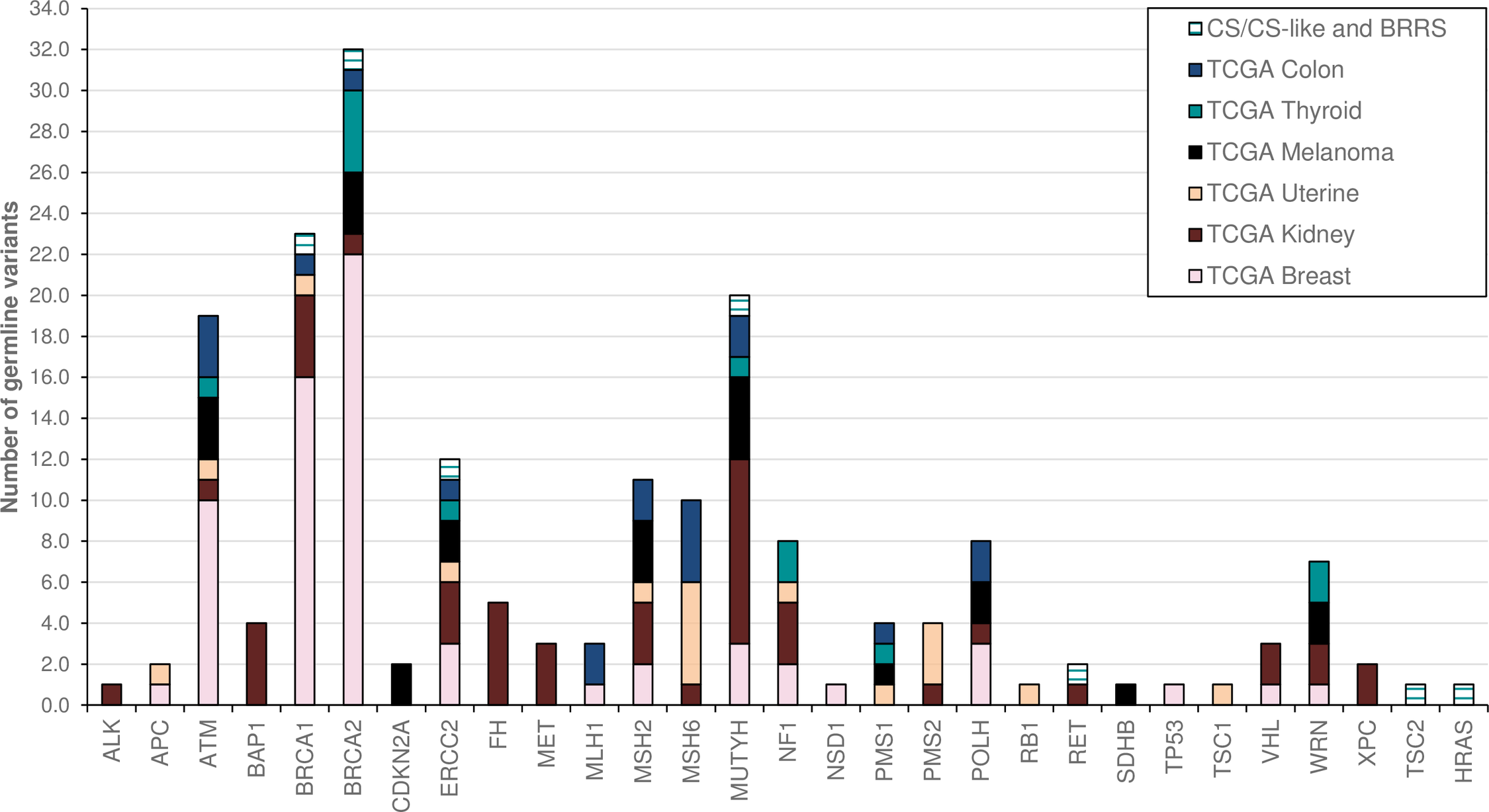

Supplement: S2 Fig — Abbreviations: BRCA, Breast Invasive Carcinoma; THCA, Thyroid Carcinoma; KIRC, Kidney Renal Clear Cell Carcinoma; KIRP, Kidney Renal Papillary Cell Carcinoma; UCEC, Uterine Corpus Endometrial Carcinoma; UCS, Uterine Carcinosarcoma; COAD, Colon Adenocarcinoma; SKCM, Skin Cutaneous Melanoma. (TIF) [file pgen.1007352.s014.tif]
